# Blood Inflammatory Cytokines as Predictors of Depression in Patients With Glioma

**DOI:** 10.3389/fpsyt.2022.930985

**Published:** 2022-06-09

**Authors:** Huayu Li, Xiaohan Shi, Fan Yang, Xinrui Zhang, Feng Li

**Affiliations:** ^1^School of Nursing and Rehabilitation, Shandong University, Jinan, China; ^2^School of Physical Education, Yantai University, Yantai, China; ^3^Department of Neurosurgery, Affiliated Cancer Hospital of Shandong First Medical University, Jinan, China

**Keywords:** glioma, neuropsychology, depression, inflammatory cytokines, clinical biomarkers

## Abstract

**Background:**

Depression commonly develops as a comorbid disorder related to glioma, which affects the patients’ physical function and prognosis. Circulating inflammatory cytokines are potential predictors of depression in disparate cancers. However, less research has specifically investigated this aspect within the context of glioma.

**Study objectives:**

The objective of this study was to investigate the occurrence of depression in patients with glioma and draw a comparison of the ability to predict it through diverse inflammatory cytokines.

**Methods:**

A total of 203 patients with stage I–IV glioma were enrolled in this study. Depression was evaluated according to the Hamilton Depression Scale, and the plasma inflammatory cytokines levels were simultaneously measured. We performed the receiver operating characteristic (ROC) analysis to confirm the abilities of identified inflammatory cytokines to predict depression.

**Results:**

Among the 203 patients with glioma, 135 (66.5%) showed obvious depressive symptoms. Proinflammatory cytokines, including interleukin (IL)-6 (area under the curve (AUC) = 0.76) and tumor necrosis factor (TNF)-α (AUC = 0.75), showed good performance in accurately predicting depression in patients with glioma. These inflammatory cytokines indicated great potential to be depression biomarkers regardless of the patients’ disparate treatment experience.

**Conclusion:**

With their relatively simple and time-saving measurement procedures, inflammatory cytokines should be seriously considered effective clinical screening and diagnostic tools, as well as potential biomarkers for depression in patients with glioma.

## Introduction

Glioma accounts for 80% of primary craniocerebral malignant tumors (1), and it has an occurrence of approximately 8/100,000 per year (2, 3). It causes general clinical symptoms such as headache (4), hemisensory loss, and seizures (5, 6). Besides, psychological disorders are also significant problems plaguing patients (7, 8). Among all psychological disorders, the most common concomitant symptom that is associated with glioma is depression (9). Evidence has shown that patients with glioma have a higher risk of depression compared with other patients with cancer (10). Depression is disruptive for glioma survivors, as it does not only detract from one’s cognitive and physical function but also influences the quality of life and even leads to the recurrence, metastasis, and deterioration of tumors (11), negatively affecting treatment and rehabilitation (12). In clinical practice, while doctors and nurses frequently focus on the neurological symptoms of patients with glioma, psychological changes tend to be ignored (13, 14). As a result, this makes it extremely difficult to accurately diagnose depression and hinders effective symptom management of patients with glioma.

In the past few years, more studies concentrated on inflammatory cytokines released by activated inflammation as mood predictors among different patients with cancer (15, 16). Bouchard and colleagues (17) discovered the proinflammatory cytokines, including interleukin-1β (IL-1β) and tumor necrosis factor-α (TNF-α), to be positively correlated with depressed mood in patients with breast cancer. Similarly, McFarland et al. (18) discovered that depressed patients with lung cancer possessed higher levels of proinflammatory cytokines compared with patients without depressive symptoms, which supports the contention that inflammatory cytokines, such as IL-6 and TNF-α, play a crucial role in the patients’ mood disorders by affecting the neurotransmitter metabolism, ultimately leading to abnormal neuroendocrine function. Consequently, it became increasingly recognized that inflammatory cytokines should be regularly taken into account as a promising screening tool for depression prediction. Du et al. (19) demonstrated that sputum IL-6 and TNF-α served as effective predictors for depressive symptoms in patients with lung cancer, achieving an area under the curve (AUC) of 0.81 and 0.72, respectively, as well as considerable sensitivity and specificity values. Although inflammatory cytokines with genetic variations were shown to have predictive capacities, it has not yet been clarified whether circulating inflammatory cytokines can predict depression in patients with glioma.

To investigate the relationship between alterations of inflammatory cytokines and depression in patients with glioma, we conducted a cross-sectional study. We enrolled 203 patients with glioma and assessed their magnitude of depression, while simultaneously measuring the levels of their plasma inflammatory cytokines. Moreover, we compared the predictive performance of inflammatory cytokines to determine sensitive, specific, simple, and timely inflammatory biomarkers, which would be of great significance to assist medical staff in early screening and diagnosis of depression and formulate precise and effective treatment strategies.

## Materials and Methods

### Participants

We recruited 203 participants diagnosed with glioma from the Neurosurgery Department of Affiliated Tumor Hospital of Shandong First Medical University between December 2020 and December 2021. The inclusion criteria were as follows: (a) 18–70 years old, (b) clear consciousness, good comprehension, and normal language expression, and (c) volunteering to participate in this research. The exclusion criteria were as follows: (a) other serious systemic diseases or history of diseases affecting the immune function, (b) recent use of antipsychotic and immunocompromised drugs, (c) cognitive dysfunction, and (d) steroid therapy or infection and fever within the last month.

Our research was approved by the Ethics Review Board of School of Nursing and Rehabilitation, Shandong University (No. 2020-R-071), prior to enrollment, and all participants signed an informed consent.

### Research Instruments

#### General Information

Demographic data and clinical characteristics, including age, sex, body mass index (BMI), pathological grade, and tumor location, were collected from the patients’ self-report or electronic medical records.

#### Depression

The occurrence and severity of depression were evaluated according to the Hamilton Depression Scale 24 (HAMD-24), which is the most widely used clinical depression reporting scale compiled by Hamilton in 1960 (20). HAMD-24 contains 7 dimensions, namely, anxiety/somatization, weight, cognitive disturbance, diurnal variation, retardation, sleep disturbance, and hopelessness. Then, the HAMD-24 score can be calculated by summing the scores of the 7 domains, and the higher the score, the more severe the depression. In this study, patients with an HAMD-24 score > 7 were regarded as to have significant depression (21), and the HAMD-24 exhibited a high test-retest reliability in our research (Cronbach’s α = 0.90).

### Laboratory Measures

#### Sample Collection

Venous blood samples (3–4 ml) were collected by venipuncture into chilled ethylenediaminetetraacetic acid (EDTA) tubes on the same day of depression evaluation and placed in a 4°C refrigerator. After centrifugation, the samples were stored in a −80°C freezer until batched assay.

#### Inflammatory Biomarkers

We analyzed the plasma concentrations of the inflammatory cytokines, including IL-1, IL-6, IL-4, IL-10, IL-18, TNF-α, and C-reactive protein (CRP), using the Human High Sensitivity Cytokine Base Kit A. Plasma concentrations of the inflammatory cytokines were measured by the polymerase chain reaction (PCR) assay, and CRP was tested by standard turbidimetric assay techniques, following to the manufacturer’s protocol (ESUN BIO, CHINA). All intra- and interassay coefficients of variation (CVs) were reliably ≤ 10%, and the data of inflammatory biomarkers were excluded if the values were below the limit of detection. All blood samples were measured in duplicate, and mean levels were used in the final results.

### Data Analysis

Demographic and clinical data were represented by descriptive statistics. Continuous variables were represented by mean and standard deviation, while categorical variables were expressed by frequency (N) and percentage (%). For continuous variables, the relationships between the general variables and inflammatory cytokines with depression were analyzed using the Student’s *t*-test or Mann-Whitney test, depending on data normality, while Fisher’s exact test or chi-square test was used for dichotomous variables. We calculated the receiver operating characteristics (ROC) curve to determine the AUC, sensitivity, and specificity, thus evaluating the depression diagnostic accuracy of those cytokines. We applied the maximum value of the Youden index (sensitivity + specificity − 1) to represent the optimal cutoff points for inflammatory cytokines. To compare AUCs, the Delong Clarke-Pearson method was used. To solve the inadequate sample size in this research and confirm the ROC accuracy, we implemented the support vector machine (SVM) algorithm to determine the ROC capability. The SVM model was considered as the promising perfect performance. The samples were randomly divided into two parts, namely, training data (75%) to build the model and testing data (25%) to assess the predictive ability. The SVM model was constructed using the e1071 package and a default value for the cost of 1 with a radial basis function kernel. In addition, three 10-fold cross-validations were used to avoid overfitting. The statistical data analysis was conducted using RStudio (4.1.1) and MedCalc (20.2.7). Graphic plotting was performed using GraphPad Prism (8.0.2). A two-tailed *P* < 0.05 was statistically significant.

## Results

### Sociodemographic Characteristics of Patients With Glioma

A total of 203 patients with glioma with an average age of 54.1 years were enrolled in our study, among which 64 (31.5%) had low-grade glioma (WHO I–II grade), and 139 (68.5%) had high-grade glioma (WHO III-IV grade). The sociodemographic characteristics of the participants are listed in Table 1.

**TABLE 1:** Comparison of demographic and clinical characteristics between glioma patients with and without depression.

Variables	*N* = 203 (%)	Non-Depression	Depression	*P*
			
		*N* = 68 (%)	*N* = 135 (%)	
**Age**				
<60y	126 (62.1)	45 (66.2)	81 (60.0)	0.392
≥60y	77 (37.9)	23 (33.8)	54 (40.0)	
**Sex**				
Male	103 (50.7)	38 (55.9)	65 (48.1)	0.298
Female	100 (49.3)	30 (44.1)	70 (51.9)	
**BMI**				
<18.5	5 (2.5)	3 (4.4)	2 (1.5)	0.069
18.5-23.9	88 (44.3)	25 (36.8)	63 (46.7)	
24.0-27.9	83 (40.9)	26 (38.2)	57 (42.2)	
≥28	27 (13.3)	14 (20.6)	13 (9.6)	
**Smoking history**				
Yes	29 (14.3)	8 (11.8)	21 (15.6)	0.466
No	174 (85.7)	60 (88.2)	114 (84.4)	
**Alcohol history**				
Yes	24 (11.8)	6 (8.8)	18 (13.3)	0.348
No	179 (88.2)	62 (91.2)	117 (86.7)	
**KPS**				
≤60	35 (17.2)	7 (10.3)	28 (20.7)	0.063
>60	168 (82.8)	61 (89.7)	107 (79.3)	
**Surgery**				
Received	139 (68.5)	57 (83.8)	82 (60.7)	0.001
Not Received	64 (31.5)	11 (16.2)	53 (39.3)	
**Radiotherapy**				
Received	73 (36)	24 (35.3)	49 (36.3)	0.888
Not Received	130 (64)	44 (64.7)	86 (63.7)	
**Chemotherapy**				
Received	75 (36.9)	17 (25.0)	58 (43.0)	0.012
Not Received	128 (63.1)	51 (75.0)	77 (57.0)	
**WHO glioma grade**				
Low(I-II)	64 (31.5)	26 (38.2)	38 (28.1)	0.144
High(III-IV)	139 (68.5)	42 (61.8)	97 (71.9)	
**Lobe**				
Temporal	31 (15.3)	7 (10.3)	24 (17.8)	0.326
Frontal	71 (35)	25 (36.8)	46 (34.1)	
Occipital	9 (4.4)	1 (1.5)	8 (5.9)	
Parietal	23 (11.3)	9 (13.2)	14 (10.4)	
Other	69 (34.0)	26 (38.2)	43 (31.9)	

*BMI, body mass index; KPS, karnofsky performance status.*

### Depressive Characteristics of the Cohort

**FIGURE 1:**
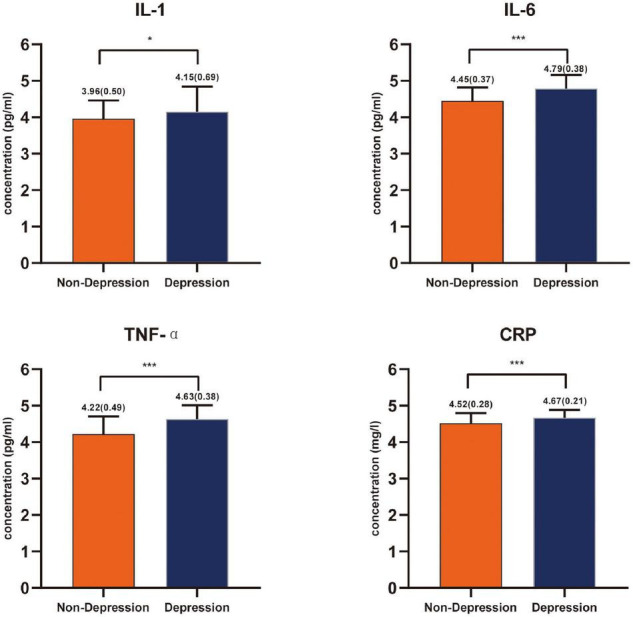
Inflammatory cytokines levels’ comparison between the non-depressed group and the depressed group in patients with glioma expressed with mean and standard deviation. *P < 0.05, ***P < 0.001.

According to the HAMD score, 68 patients with a score ≤ 7 (mean, 3.24; *SD*, 2.14) were classified into the non-depression group, while the remaining 135 patients with a score > 7 (mean, 17.35; *SD*, 8.70) were classified into the depression group. Striking statistically significant differences were observed between the two groups in the surgery (*P* = 0.001) as well as chemotherapy (*P* = 0.012) (Table 1). Compared with the non-depression group, patients with depressive symptoms had higher levels of inflammatory cytokines, including IL-1, IL-6, TNF-α, and CRP (Figure 1).

### The Performance of Inflammatory Cytokines in Predicting Depression

**FIGURE 2:**
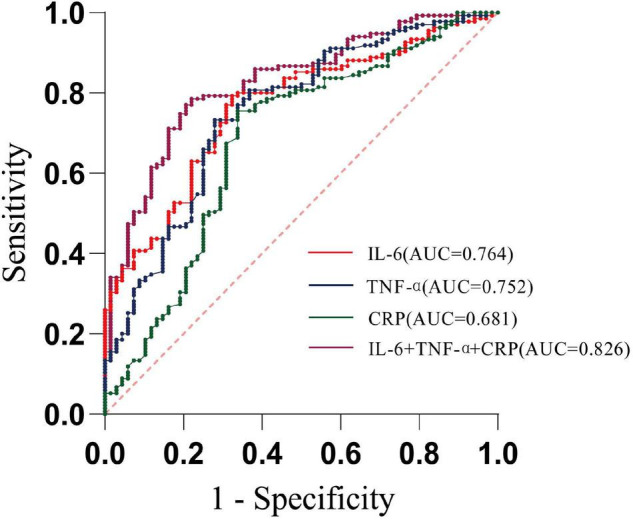
Receiver operating characteristic curve analysis predicting depression using circulating inflammatory cytokines. (*AUC*—area under curve; dotted line—reference line).

We performed the ROC analysis to confirm a precise threshold for various inflammatory cytokines forecasting depression. Table 2 lists the results for the predictive performance of 7 individual inflammatory biomarkers. The best predictors of depressive symptoms were IL-6 (AUC: 0.76, 95% CI: 0.70–0.83), TNF-α (AUC: 0.75, 95% CI: 0.68–0.82), and CRP (AUC: 0.68, 95% CI: 0.60–0.76) (Figure 2). Then, algorithm development generated multiple prospective combinations of inflammatory biomarkers. We chose the “Linear Model – Log Value – three Biomarkers” connection of IL-6, TNF-α, and CRP to calculate AUC of the model including the three inflammatory cytokines, which manifested a preferable predictive power yielding an AUC of 0.83, and was higher than all individual biomarkers, as shown in Table 2 and Figure 2. To determine the accuracy and stability of the ROC models, SVM models were constructed and evaluated. The AUCs obtained from the SVM model were 0.77 for IL-6, 0.84 for TNF-α, and 0.62 for CRP, which supported the exceptional prediction performance for depressive symptoms of the inflammatory cytokines, especially IL-6 and TNF-α.

**TABLE 2:** Performance of depression prediction using inflammatory cytokines.

Variables	*AUC*	95%*CI*	Cut-off	Sensitivity/Specificity (%)
IL-6	0.764	0.697-0.831	4.590	79.3/67.7
TNF-α	0.752	0.681-0.824	4.521	73.3/72.1
CRP	0.681	0.599-0.763	4.566	75.6/66.2
IL-6 + TNF-α + CRP	0.826	0.767-0.884	4.378	78.5/77.9
IL-1	0.556	0.475-0.637	−	−
IL-18	0.526	0.440-0.612	−	−
IL-4	0.503	0.417-0.590	−	−
IL-10	0.392	0.205-0.479	−	−
Age	0.555	0.472-0.639	−	−
BMI	0.431	0.345-0.517	−	−

*IL, interleukin; TNF, tumor necrosis factor; CRP, C-reactive protein; BMI, body mass index.*

According to the Youden index maximum values, it was found that when the cutoff value for IL-6 was 4.590 pg/ml, the sensitivity was 79.3% and the specificity was 67.7%, while when the cutoff value for TNF-α was 4.521 pg/ml, the sensitivity and specificity were 73.3 and 72.1%, respectively (Table 2). In addition, using IL-6 as the reference, we drew a comparison of the AUCs between different inflammatory cytokines and found no statistically significant discrepancy between IL-6 and TNF-α (*P* = 0.641).

### The Predictive Abilities of Inflammatory Biomarkers in Patients With Different Clinical Characteristics

Significant differences were observed in the operation (*P* = 0.001) and chemotherapy (*P* = 0.012) between the depression and non-depression groups, as depicted in Table 1. We further observed that the TNF-α and CRP levels in patients undergoing surgery were higher than those without surgery, (*P* = 0.002, *P* < 0.001, respectively), while patients receiving chemotherapy showed higher IL-6 compared with the non-chemotherapy group (*P* = 0.001). We performed ROC analysis to distinguish the depression of patients with different clinical characteristics according to the IL-6, TNF-α, and CRP values. The comparison of depressed and non-depressed patients with disparate therapeutic methods determined by the cutoff values of three inflammatory cytokines levels is presented in Supplementary Files 1–4, and statistically significant differences in the AUC produced by three cytokines were observed in the group without surgery (IL-6 and TNF-α, *P* = 0.031; TNF-α and CRP, *P* = 0.007; IL-6 and CRP, *P* = 0.025, respectively).

## Discussion

In this work, we reported the consequences for a cross-sectional study assessing the plasma inflammatory biomarkers levels, as supplementary means in the inchoate screening and diagnosis for depression in patients with glioma. Our research revealed that inflammatory cytokines satisfy the criteria for predicting depression in patients with glioma, both individually and in combination. Specifically, IL-6 and TNF-α demonstrated a favorable performance prediction.

### Depression in Patients With Glioma

In our glioma cohort, the incidence of depression was 66.5%, which is higher than in Jiao’s report (22) of 45.5%. We speculate that it was potentially caused by investigating using different scale measurements. Generally speaking, clinicians frequently blame depression on disease or treatment (23), which indicates that depressive symptoms in patients with glioma are underdiagnosed and do not obtain effective treatment. It has not yet been asserted that randomized controlled trials demonstrated the efficiency of antidepressants for depressive symptoms in patients with glioma (24), which suggests that we should concentrate on confirming the biomarkers closely linked with depression and make further efforts to identify early depressive symptoms. Hence, individualized psychological intervention measures can be developed, and the patients’ quality of life can be improved.

### Inflammatory Biomarkers Effectively Predict Depression

This research, to our best information, represented the first study applying the ROC analyses to evaluate the availability of inflammatory cytokines as predictors of depressive symptoms in patients with glioma. Our findings showed that depressed patients had marginally greater levels of inflammatory conditions than those without depression, and multifarious cytokines were activated, containing IL-1, IL-6, TNF-α, and CRP, which was harmonious with the conclusions of accessible inflammation research in other cancer types (17, 25, 26). Cytokines IL-6 and TNF-α appeared to be good predictors for depression. Moreover, among the detected inflammatory cytokines, IL-6 showed to be the most effective screening tool to predict depression, with an AUC of 0.76, a sensitivity of 79.3%, and a specificity of 67.7%, followed by TNF-α with an AUC of 0.75. A combination of elevated IL-6, TNF-α, and CRP provided preferable prediction performance in terms of AUC (0.83) over individual biomarkers, with considerable sensitivity (78.5%) and specificity (77.9%) for depression, but it demanded higher costs and additional time. Albeit, this was the first report in patients with glioma to illustrate a satisfactory effect for inflammatory cytokines in predicting depression, which was consistent with the previous discoveries of studies in diverse diseases. For instance, Ho et al. (27) proposed that circulating cytokines IL-2 (AUC, 0.78; sensitivity, 86.7%; specificity, 52.9%) and IL-5(AUC, 0.76; sensitivity, 66.7%; specificity, 80.9%) showed great performance in predicting depression in breast cancer. Furthermore, Wesley et al. (28) reported that the circulating cytokine CRP (AUC, 0.73; sensitivity, 73.0%; specificity, 64.0%) demonstrated notable prediction ability in moderate/severe obstructive sleep apnea (OSA). It is worth mentioning that the good prediction performance of inflammatory biomarkers on depression could still be observed in spite of whether the patients had undergone surgery or chemotherapy in our study.

### The Complicated Mechanisms Underlying Inflammatory Cytokines Triggering Depression

An explanation for the elevated inflammatory cytokines levels in patients with glioma was presumably that the tissue destruction caused by surgery, radiotherapy, and chemotherapy could trigger damage-associated molecular patterns (DAMPs), which are combined with pattern recognition receptors (PRRs) on white blood cells, especially macrophages, eventually leading to expressing the nuclear factor-κβ (NF-κβ) and releasing multitudinous cytokines (29), containing IL-6 and TNF-α (30). Further evidence has also proposed IL-6 to be in a position to stimulate the hypothalamic-pituitary-adrenal (HPA) axis and activate it (31), altering the production, metabolism, and transportation of neurotransmitters, which synergistically affected mood. In addition, IL-6 caused an increase in glucocorticoids and the activation of tryptophan 2, 3-dioxygenaes (TDO) (32); this resulted in lower levels of serotonin (5-HT) in the brain (33, 34), which has been confirmed to be closely correlated with depression (32). The most noteworthy observation was that 5-HT receptors were discovered in glioma cells, and 5-HT could regulate the proliferation, migration, and invasion of glioma cells *in vitro* (35).

Plenty of studies convincingly manifested that elevated TNF-α levels existed in patients with major depressive disorder (36–38). TNF-α was able to increase the permeability of the blood-brain barrier (BBB) (39, 40), such that dysfunctional BBB has been asserted to speed up the infiltration of inflammatory mediators and stimulate peripheral immune cells to enter into the central nervous system; consequently, behavioral abnormalities and mood disorders were triggered (41). TNF-α also enhanced the capacity of 5-HT and norepinephrine (NA) reuptake transporters, effectively reducing the synaptic concentrations of 5-HT and NA *via* the activation of p38 mitogen-activated protein kinase (MAPK), consequently contributing to depressive symptoms (34, 42).

C-reactive protein is an acute phase protein generated by the liver in response to innate immune cytokines, especially IL-6 and TNF-α, and it possesses both pro- and anti-inflammatory effects (43, 44). CRP has also been shown to be capable of activating neutrophils and monocytes, promoting TNF-α secretion in a feedback way (45). Given the prominence of CRP in psychiatry as an emerging multipurpose inflammatory biomarker, it has been considered for routine measurement in medical centers and research laboratories (46).

It is noteworthy that in our results, IL-10 produced a small AUC (0.392) for depression prediction, but the 95% CI value did not include 0.5. After eliminating the reason that multicollinearity (tolerance, 0.694 to 0.871; variance inflation factors, 1.148–1.441) among all identified variables would lead to deviation in the results, we speculated that IL-10 possibly played a significant role in depression in patients with glioma. IL-10 serves as an anti-inflammatory cytokine, and it is classically related to protective functions in the central nervous system in different neurodegenerative and neuroinflammatory conditions (47). Clinical and animal research revealed that increased IL-6 and decreased IL-10 would result in depression (48, 49). It was indeed probable that, when the levels of proinflammatory cytokines are extremely high, the production and beneficial effects of IL-10 would be suppressed.

In summary, coincident patterns of inflammatory cytokines contributed to inflammatory-related reactions despite the varied expression levels of inflammatory cytokines in various diseases. Due to a range of peculiarities of inflammatory cytokines, they showed great potential to be used as a clinical screening instrument for depressive symptoms in patients with glioma.

## Limitation

Our research has several limitations. First, the depression assessment was based on a scale rather than professional diagnosis. Therefore, the generalization to clinical depression was probably restricted. Second, this cross-sectional research with relatively modest samples was incapable of providing rigorous control of baseline inflammatory biomarkers and psychosocial factors. Thus, our speculations need to be considered with caution. In future research, further exploration in longitudinal studies with larger samples is warranted to validate our findings.

## Conclusion

Peripheral immune dysfunction presumably plays a crucial role in weakening the functional and structural changes in the brain that underlie the pathophysiology of depression. Hence, peripheral inflammatory mediators with their simple, feasible, efficient, and economical detection methods have emerged as potential biomarkers to predict the onset of depression. In this research, relatively stable markers, such as IL-6 and TNF-α, demonstrated enhanced and robust performance in predicting depression in patients with glioma. This indicates that the concurrent elevation of IL-6 and TNF-α should be highly suspected of depression, while meanwhile, reminding the clinical staff to carefully observe and timely formulate innovative therapeutic strategies.

## Data Availability Statement

The raw data supporting the conclusions of this article will be made available by the authors, without undue reservation.

## Ethics Statement

The studies involving human participants were reviewed and approved by Ethics Review Board of School of Nursing and Rehabilitation, Shandong University. The patients/participants provided their written informed consent to participate in this study.

## Author Contributions

FL: conceptualization, project administration, funding acquisition, and writing – review and editing. HL: data collection, software, formal analysis, and writing. XS: data collection supervision, data sorting, and writing assisting. FY: data collection supervision, software, and methodology. XZ: data collection and methodology supervision. All authors approved the final manuscript for submission.

## Conflict of Interest

The authors declare that the research was conducted in the absence of any commercial or financial relationships that could be construed as a potential conflict of interest.

## Publisher’s Note

All claims expressed in this article are solely those of the authors and do not necessarily represent those of their affiliated organizations, or those of the publisher, the editors and the reviewers. Any product that may be evaluated in this article, or claim that may be made by its manufacturer, is not guaranteed or endorsed by the publisher.
