# Supplementary material for: Blood Inflammatory Cytokines as Predictors of Depression in Patients With Glioma
Source: Front Psychiatry. 2022 Jun 9;13:930985. doi: 10.3389/fpsyt.2022.930985 (PMC9218211; doi:10.3389/fpsyt.2022.930985)
Supplement: Supplementary file 1 [file Data_Sheet_1.docx]

**Blood inflammatory cytokines as predictors of depression in patients with glioma**

Huayu Li, Xiaohan Shi, Fan Yang, Xinrui Zhang, Feng Li

**Supplement1:** The receiver operating characteristic curve(ROC)for distinguishing depressed patients who have not received surgery based on inflammatory biomarkers values. The sensitivities of IL-6, TNF-α and CRP were 75.5% (95%CI: 62.4-85.1), 81.1% (95%CI: 68.6-89.4) and 50.9% (95%CI: 37.9-63.9), respectively, and the specificities were 81.8% (95%CI: 52.3-96.8), 72.7% (95%CI: 43.4-90.3), and 72.7% (95%CI: 43.4-90.3), respectively. The concentration limits of IL-6, TNF-α, and CRP were 4.610 pg/mL (Youden Index :0.573), 4.525 pg/mL (Youden Index :0.539), 4.715 mg/ L (Youden Index :0.237), respectively.


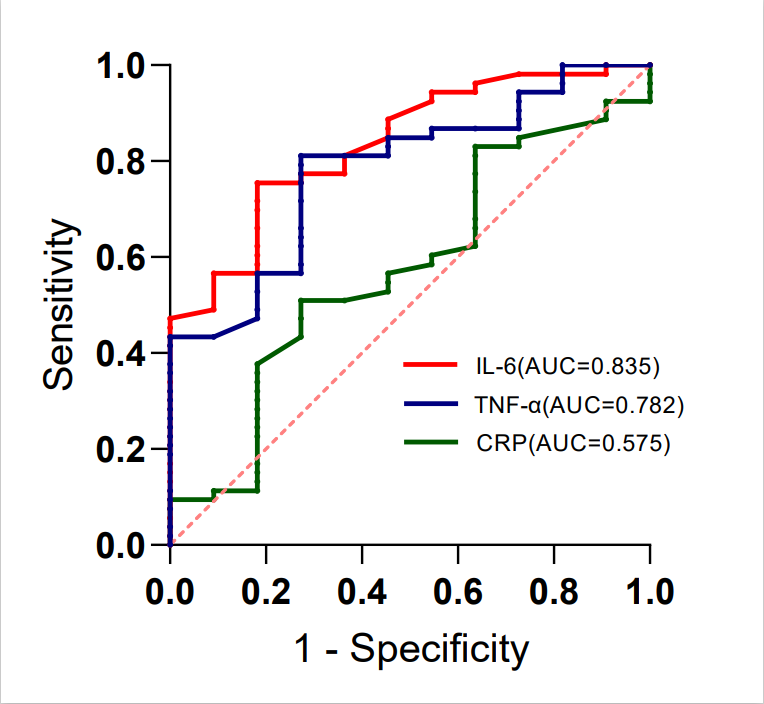


**Figure S1**. ROC analysis for distinguishing depression of patients without received surgery

**Supplement2:** The receiver operating characteristic curve(ROC)for distinguishing depressed patients who have received surgery based on inflammatory biomarkers values. The sensitivities of IL-6, TNF-α and CRP were 80.5% (95%CI: 70.6-87.6), 78.1% (95%CI: 68.0-85.6) and 72.0% (95%CI: 61.4-80.5), respectively, and the specificities were 64.9% (95%CI: 51.9-76.0), 64.9% (95%CI: 51.9-76.0), and 71.9% (95%CI: 59.2-81.9), respectively. The concentration limits of IL-6, TNF-α, and CRP were 4.585 pg/mL (Youden Index :0.454), 4.430 pg/mL (Youden Index :0.430), 4.565 mg/ L (Youden Index :0.439), respectively.


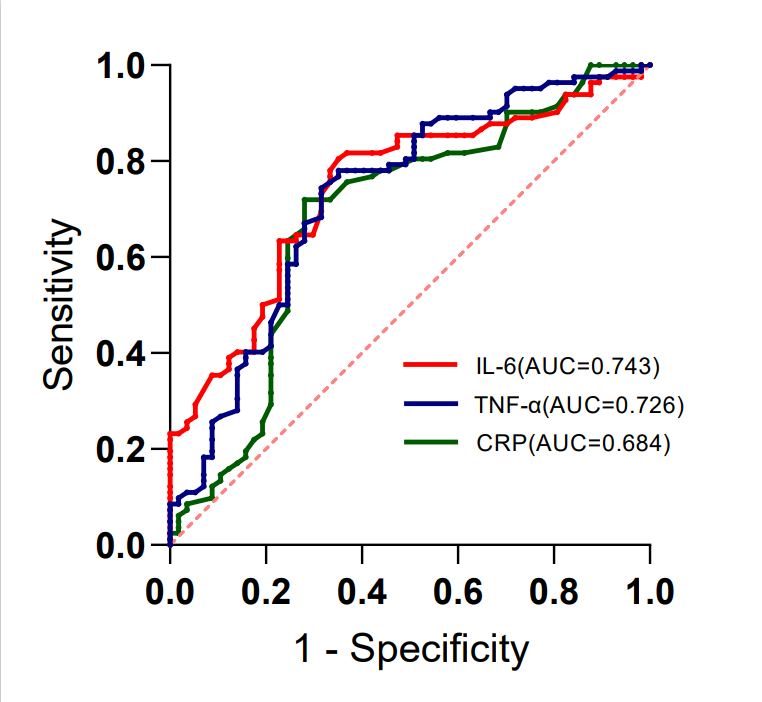


**Figure S2**. ROC analysis for distinguishing depression of patients with received surgery

**Supplement3:** The receiver operating characteristic curve(ROC)for distinguishing depressed patients who have not received chemotherapy based on inflammatory biomarkers values. The sensitivities of IL-6, TNF-α and CRP were 68.8% (95%CI: 57.8-78.1), 72.7% (95%CI: 61.9-81.4) and 74.0% (95%CI: 63.3-82.5), respectively, and the specificities were 70.6% (95%CI: 57.0-81.3), 64.7% (95%CI: 51.0-76.4), and 58.8% (95%CI: 45.2-71.2), respectively. The concentration limits of IL-6, TNF-α, and CRP were 4.610 pg/mL (Youden Index :0.394), 4.525 pg/mL (Youden Index :0.374), 4.565 mg/ L (Youden Index :0.329), respectively.


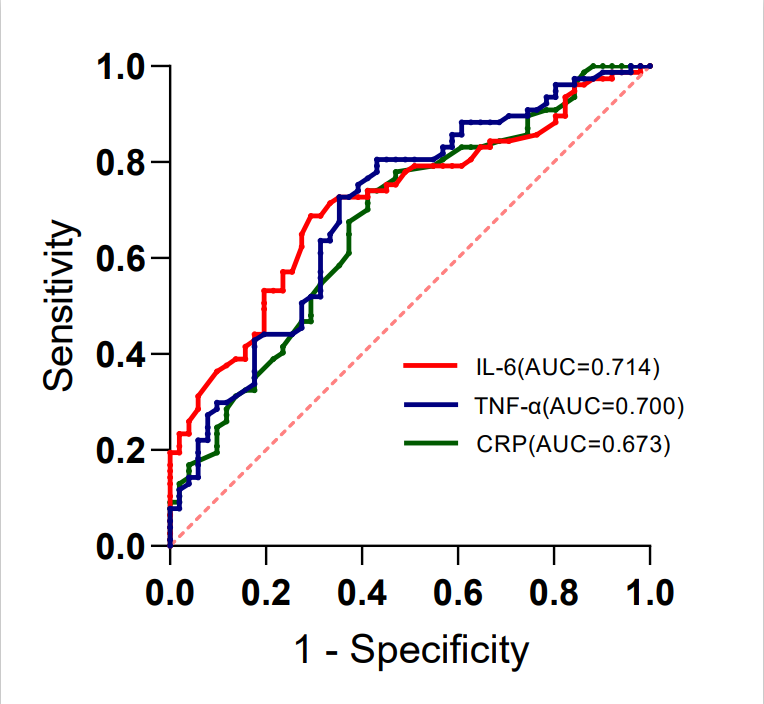


**Figure S3**. ROC analysis for distinguishing depression of patients without received chemotherapy

**Supplement4:** The receiver operating characteristic curve(ROC) for distinguishing depressed patients who have received chemotherapy based on inflammatory biomarkers values. The sensitivities of IL-6, TNF-α and CRP were 89.7% (95%CI: 79.2-95.2), 72.4% (95%CI: 59.8-82.2) and 77.6% (95%CI: 65.3-86.4), respectively, and the specificities were 64.7% (95%CI: 41.3-82.7), 94.1% (95%CI: 73.0-99.7), and 88.2% (95%CI: 65.7-97.9), respectively. The concentration limits of IL-6, TNF-α, and CRP were 4.580 pg/mL (Youden Index :0.544), 4.535 pg/mL (Youden Index :0.665), 4.565 mg/ L (Youden Index :0.658), respectively.


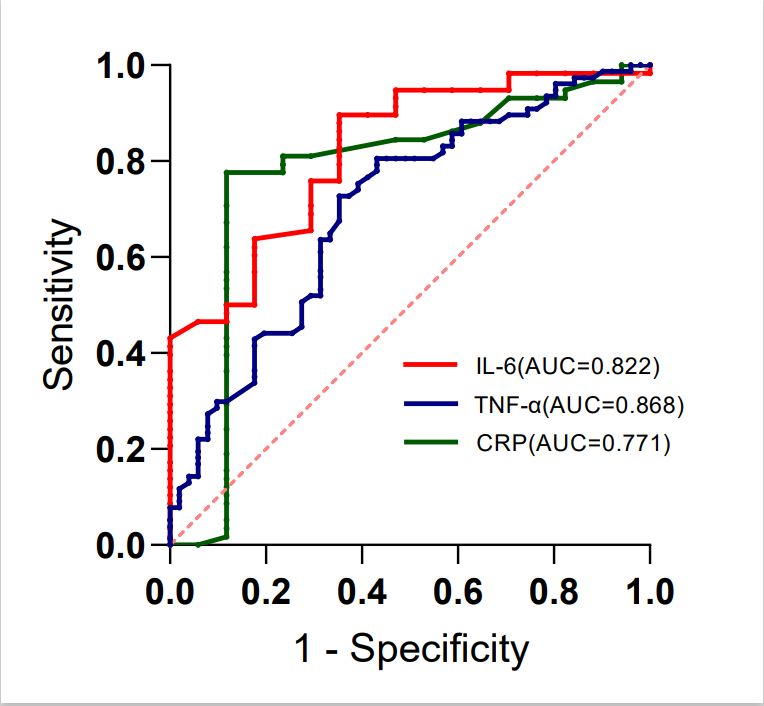


**Figure S4**. ROC analysis for distinguishing depression of patients with received chemotherapy
